# SPOUSAL DRIVING CESSATION AND VOLUNTEERING: MODERATING ROLE OF CAREGIVING AND DEPRESSION

**DOI:** 10.1093/geroni/igz038.3441

**Published:** 2019-11-08

**Authors:** Angela L Curl, Christine M Proulx, Teresa M Cooney

**Affiliations:** 1 Miami University, Oxford, Ohio, United States; 2 University of Missouri, Columbia, Missouri, United States; 3 University of Colorado Denver, Denver, Colorado, United States

## Abstract

Driving cessation is a normative transition in later adulthood, yet previous research shows that having a spouse who ceases to drive, even when you are still able to, negatively impacts one’s own engagement (i.e., formal and informal volunteering). Little is known about the conditions under which volunteer engagement might vary after a spouse stops driving. We used longitudinal data from 10 waves (1998–2016) of the Health and Retirement Study (HRS) to examine whether depressive symptoms and caregiving demands moderate the association between a spouse’s driving cessation and one’s own formal and informal volunteering. Respondents were included if, at baseline, both spouses participated in HRS, both were age 65+, and both were still driving. Respondents were dropped at the time of their own driving cessation, to focus specifically on the impact of spousal driving cessation. Multilevel model results for 1,370 husbands and 1,368 wives show that moderation occurred only for wives who were still driving. After controlling for sociodemographic factors, physical health, and cognitive ability, husbands’ driving cessation negatively impacted formal volunteering but only for wives who were primary ADL and IADL caregivers for their spouses. Further, husbands’ driving cessation negatively impacted informal volunteering for wives who reported relatively high levels of depressive symptoms. Results suggest the importance of contextual factors like caregiving engagement/needs and psychological wellbeing, especially for wives, when examining the role of spousal driving cessation in partners’ volunteer engagement, and highlight the need for additional research on the relationship between spousal driving cessation and volunteering for husbands.

